# Treatment outcomes of **Acinetobacter baumannii**-associated pneumonia and/or bacteraemia at the intensive care unit of Universitas Academic Hospital, Bloemfontein, South Africa

**DOI:** 10.7196/AJTCCM.2021.v27i1.122

**Published:** 2021-03-09

**Authors:** S D Maasdorp, S Potgieter, E Glover, G Joubert

**Affiliations:** 1 Divisions of Pulmonology and Critical Care, Departments of Internal Medicine and Surgery, Faculty of Health Sciences, University of the Free State, Bloemfontein, South Africa; 2 Division of Infectious Diseases, Department of Internal Medicine, Faculty of Health Sciences, University of the Free State, Bloemfontein, South Africa; 3 Department of Biostatistics, Faculty of Health Sciences, University of the Free State, Bloemfontein, South Africa

## Abstract

**Background:**

Nosocomial infection with multidrug-resistant (MDR) *Acinetobacter* *baumannii* is associated with high mortality rates and the optimal treatment regimen is uncertain.

**Objectives:**

To compare outcomes, as well as ICU and in-hospital survival rates of patients with *A. baumannii* pneumonia and/or bacteraemia who were treated with colistin monotherapy v. colistin/tigecycline combination therapy

**Methods:**

This was a retrospective cross-sectional study of patients admitted to the multidisciplinary ICU of Universitas Academic Hospital, Bloemfontein, South Africa, between 1 January 2018 and 31 December 2019.

**Results:**

Sixteen patients were included in the study. Nine patients were treated with a combination of colistin and tigecycline, while 7 patients were treated with colistin only. Seven out of 9 (77.8%) patients in the colistin/tigecycline combination therapy group were treated successfully and survived until discharge from ICU, as opposed to 2 out of 7 (28.6%) in the colistin monotherapy group (relative risk (RR) 2.7; 95% CI 0.80 - 9.24). Five out of 9 (55.6%) in the colistin/tigecycline combination therapy group v. 2 out of 7 (28.6%) in the colistin monotherapy group survived until discharge from hospital (RR 1.94; 95% CI 0.53 - 7.20).

**Conclusion:**

Although ICU survival in patients with *A. baumannii* infection was better when treated with colistin/tigecycline combination therapy compared with colistin monotherapy, a statistically significant difference could not be detected. Adequately powered prospective clinical trials are required to detect statistically significant differences in treatment outcomes.

## Background

Nosocomial infection with multidrug-resistant (MDR) *Acinetobacter* *baumannii* is a global problem and is associated with high mortality rates.^[1,2]^ The optimal antibiotic regimen for treating MDR A. *baumannii* is largely unknown. Tigecycline-based therapy has been associated with higher mortality and treatment failure rates than colistin-based treatment regimens.^[3]^ Although combinations of antibiotics are often used when treating resistant organisms, a study conducted by Amat *et al*.^[4]^ found no mortality benefit in using a combination of colistin and tigecycline v. colistin monotherapy. The lack of benefit in their study may, however, have been influenced by using standard manufacturer-prescribed dosages of tigecycline, which are lower than the current recommended dosages.^[5–7]^ Despite lack of evidence, international consensus guidelines still recommend the use of colistin in combination with a second antimicrobial agent to which the organism may be susceptible.^[8]^ Complicating the management of MDR *A. baumannii* further is that colistin drug sensitivity testing is particularly unreliable using automated platforms. The Vitek2 (bioMérieux, USA), used by the National Health Laboratory Services (NHLS) laboratory at our institution, significantly underestimates colistin resistance. This may result in inappropriate treatment, especially when colistin monotherapy is used.^[9]^ Review of the antibiotic susceptibility profiles of *A. baumannii* cultured from clinical isolates in the intensive care unit (ICU) at Universitas Academic Hospital (UAH) in Bloemfontein, South Africa (SA), revealed that these isolates were susceptible only to colistin and tigecycline, and otherwise resistant to all other antibiotic classes, including carbapenems. Whereas colistin monotherapy was our preferred agent for clinically significant *A. baumannii* infection prior to 2019, we opted for combination therapy with colistin and tigecycline midway through 2019 in view of an impression of poor clinical outcomes with colistin monotherapy. The aim of the present study was to compare outcomes of patients with *A. baumannii* infection treated with colistin monotherapy v. those treated with colistin/tigecycline combination therapy in the multidisciplinary ICU at UAH. The primary objectives were: to compare outcomes of patients with *A. baumannii* pneumonia and/or bacteraemia who were treated with colistin monotherapy v. colistin/tigecycline combination therapy; to compare ICU survival rates of patients with *A. baumannii* infection treated with colistin monotherapy v. colistin/tigecycline combination therapy; and to compare in-hospital survival rates of patients with *A. baumannii* infection treated with colistin monotherapy v. colistin/tigecycline combination therapy.

## Methods

The study was a retrospective cross-sectional study. It included patients who were diagnosed with and treated for *A. baumannii* hospital-acquired pneumonia and/or bacteraemia with either colistin or colistin and tigecycline in the multidisciplinary ICU of UAH between 1 January 2018 and 31 December 2019. UAH is a 636-bed hospital and serves as a tertiary referral centre for the Free State and Northern Cape provinces of SA, as well as Lesotho. The multidisciplinary ICU is a six-bed unit with ~30 patient admissions per month. The study was limited to the period 2018 - 2019, since an increase in the incidence of MDR *A. baumannii* infections requiring treatment with colistin or colistin/tigecycline therapy occurred during this time frame. Patients who received colistin as the sole antimicrobial agent intravenously only or via both the intravenous and inhalation routes were considered to be on colistin monotherapy. The following patients were excluded: patients deemed to have been colonised with, but not having, clinically significant *A. baumannii* infection (colonisation was inferred if the patient did not have any signs of infection such as fever, elevated white cell counts, raised C-reactive protein or procalcitonin levels and had not been initiated onto treatment for *A. baumannii* by the treating clinician at the time of culture or when culture results were available); sites of primary infection other than lung or bloodstream; patients who survived less than 24 hours after initiating antibiotic treatment with either colistin or colistin/tigecycline combination therapy, since these patients would most likely have been deemed too ill to benefit from treatment with antibiotics; neutropenic patients in view of poor overall survival rates of this particular group of patients in the unit; and incomplete or missing hospital data, where the primary objectives of clinical cure, ICU survival and in-hospital survival could not be determined because of inadequate clinical record-keeping.

Patients who were treated with either colistin monotherapy or colistin/tigecycline combination therapy for *A. baumannii* during 2018 - 2019 were identified by reviewing the medication dispensing records at Universitas Academic Hospital’s pharmacy. The investigators reviewed such identified patients’ medical records, filed at the Department of Critical Care or at Universitas Academic Hospital’s archives, as well as the Universitas Academic Hospital’s Meditech electronic health record system to determine which of these patients fulfilled the inclusion and exclusion criteria. Files of all patients admitted to the ICU are kept in a filing cabinet at the Department of Critical Care. Study data were collected and managed using REDCap (Research Electronic Data Capture; Vanderbilt University, USA) electronic data-capture tools hosted at the University of the Free State. REDCap is a secure, web-based software platform designed to support data capture for research studies, providing an intuitive interface for validated data capture; audit trails for tracking data manipulation and export procedures; automated export procedures for seamless data downloads to common statistical packages; and procedures for data integration and interoperability with external sources.^[10,11]^ Statistical analysis was performed by the Department of Biostatistics using SAS version 9.4 (SAS, USA). Standard deviations or medians and percentiles were calculated for numerical data. Frequencies and percentages were calculated for categorical data. Appropriate statistical tests (Kruskal-Wallis, *t*-tests, χ² and/or Fisher’s exact at 5% significance level) were performed and 95% confidence intervals calculated. Approval to conduct the study was obtained from the Health Sciences Research Ethics Committee of the University of the Free State (ref. no. UFSHSD2020/0029/2502) and the Free State Province Department of Health.

## Results

A total of 16 patients fulfilled the inclusion criteria and were eligible for the study (Table 1). Nine patients were treated with a combination of colistin and tigecycline, whereas 7 patients were treated with colistin only. The median age of the colistin/tigecycline group was 39 years v. 40 years for the colistin monotherapy group. The median APACHE-II score for the colistin/tigecycline group was 17 v. 12 for the colistin monotherapy group. The median SOFA score for the colistin/ tigecycline group was 4.5 v. 6 in the colistin monotherapy group. Seven out of 9 (77.8%) in the colistin/tigecycline combination therapy group were treated successfully and survived until discharge from ICU, as opposed to 2 out of 7 (28.6%) in the colistin monotherapy group (relative risk (RR) 2.7; 95% CI 0.80 - 9.24). Five out of 9 (55.6%) in the colistin/tigecycline combination therapy group v. 2 out of 7 (28.6%) in the colistin monotherapy group survived until discharge from hospital (RR 1.94; 95% CI 0.53 - 7.20). The mean duration of ventilation was 14 days for the colistin/tigecycline combination therapy group, as opposed to 17 days for the colistin monotherapy group.

## Discussion

*Acinetobacter* infections typically occur in patients who have prolonged ICU stay, are on ventilatory support, have indwelling devices and have open surgical wounds. One of the most comprehensive studies on *A. baumannii* infection in African ICUs was conducted by Ntusi *et al*. ^[12]^ in adult ICUs in Cape Town, where the investigators found that older age, HIV with a low CD4 cell count, surgery prior to ICU admission, co-morbid Gram-negative sepsis, high APACHE-II scores, multi-organ dysfunction syndrome and positive blood cultures for *A. baumannii* were all associated with increased mortality. In comparison, our study was conducted in a single multidisciplinary ICU where patients from various disciplines were admitted, as noted in Table 1. The study population was therefore fairly heterogenous. The patients included in our study were relatively young, since limited ICU resources often require prioritising better prognostic candidates and triaging of patients at the time of admission. Mortality rates of 18.9%^[13]^ and 26.5 per 100 person years^[12]^ were reported from Tygerberg and Groote Schuur hospitals, the largest tertiary hospitals in Cape Town, respectively. This is similar to the mortality rate of 22.2% of patients treated with a combination of colistin and tigecycline in our study, but markedly lower than the 71.4% mortality we found in the colistin monotherapy group. In contrast to the study by Ntusi *et al*.,^[12]^ treatment outcomes in our study were unlikely to have been influenced by comorbidities, since the prevalence of comorbidities was low, with no difference noted between the two groups. The number of ventilation days (14 v. 17 days) were lower in the combination therapy group compared with the colistin monotherapy group. The difference was however not significant. The increasing resistance of *A. baumannii* to most classes of antibiotics is as a result of this organism being highly capable of acquiring resistance mechanisms such β-lactamases, aminoglycoside-modifying enzymes, efflux pumps, porin defects and target modification.^[14]^ Moreover, reports of clonal expansion of colistin-resistant *A. baumannii* in SA have already emerged, ultimately rendering colistin, which is one of the only antibiotic options available for MDR *A. baumannii*, ineffective in treating these infections.^[15]^ MDR *A. baumannii* has emerged as an important pathogen in ICUs, with increased mortality and cost associated with the management thereof. It is therefore important to establish the most effective antibiotic treatment regimen for this pathogen, while concomitantly adhering to strict infection prevention and control measures to prevent the spread of this organism in the healthcare environment.

### Study limitations

This study has several limitations. The retrospective nature of the study may have resulted in bias in the inclusion and exclusion of patients into the study. Review of patient files for inclusion into the study was conducted by SDM and SP, with consensus reached on all patients to be included or excluded, thereby limiting selection bias for the study. Missing or incomplete data may have influenced the accuracy and reliability of the study. This was addressed by reviewing both written and electronic health records to collect and confirm as much of the required data as possible. The study, furthermore, included a very small number of study participants, since the study was limited to a 2-year period. The small number of study participants limits the power of the study to identify real differences in the treatment outcomes of the different treatment regimens.

## Conclusion

ICU survival in patients with *A. baumannii* infection was better when treated with colistin/tigecycline combination therapy compared with colistin monotherapy. The current study was, however, underpowered and larger prospective clinical trials are required to detect statistically significant differences in treatment outcomes.

## Figures and Tables

**TABLE 1:** Study results (N=16)

	**Colistin plus tigecycline** (*n*=9), *n*(%)*	**Colistin only** (*n*=7), *n*(%)*	***p*-value^†^**
Age, median (IQR)	39 (29 - 58)	40 (29 - 63)	0.49 (–32 - 20)
APACHE-II, median (IQR)	17 (13 - 20)	12 (8 - 20)	0.31 (–3 - 10)
SOFA, median (IQR)	4.5 (3 - 10)	6 (3 - 10)	0.91 (–5 - 5)
Referring discipline			
Neurosurgery	3 (33.3)	2 (28.6)	
Neurology	1 (11.1)	3 (42.9)	
General surgery	2 (22.2)	0	
Internal medicine	2 (22.2)	2 (28.6)	
Trauma	1 (11.1)	0	
Comorbidities			
HIV	2 (22.2)	1 (14.3)	1.00
Diabetes mellitus	2 (22.2)	2 (28.6)	1.00
Chronic kidney disease	1 (11.1)	0	1.00
Chronic lung disease	0	2 (28.6)	0.18
Post general surgery	1 (11.1)	0	1.00
Hypertension	1 (11.1)	3 (42.9)	0.26
Ischaemic heart disease	0	1 (14.3)	0.44
Other	0	2 (28.6)	0.18
Source of infection			0.80
Lungs	5 (55.6)	4 (57.1)	
Bloodstream	1 (11.1)	2 (28.6)	
Lungs and bloodstream	3 (33.3)	1 (14.3)	
Treatment outcome			0.13
Survived	7 (77.8)	2 (28.6)	
Died	2 (22.2)	5 (71.4)	
ICU outcome			
Survived	7 (77.8)	2 (28.6)	0.13
Hospital outcome			
Survived	5 (55.6)	2 (28.6)	0.36
Ventilation days, median (IQR)	14 (10 - 22)	17 (12 - 31)	0.29 (–20 - 8)
ICU days, median (IQR)	18 (15 - 30)	18 (12 - 32)	0.92 (–17 - 18)

CI = confidence interval

IQR = interquartile range

APACHE II = Acute Physiology and Chronic Health Evaluation II

SOFA = Sequential Organ Failure Assessment

ICU = intensive care unit

* Unless otherwise specified

^†^ 95% confidence intervals shown for median differences
